# Supplementary material for: MolNetEnhancer: Enhanced Molecular Networks by Integrating Metabolome Mining and Annotation Tools
Source: Metabolites. 2019 Jul 16;9(7):144. doi: 10.3390/metabo9070144 (PMC6680503; doi:10.3390/metabo9070144)
Supplement: Supplementary file 1 [file metabolites-09-00144-s001.zip › Supplementary Materials/Supplementary Materials/Figure_S3.pdf]

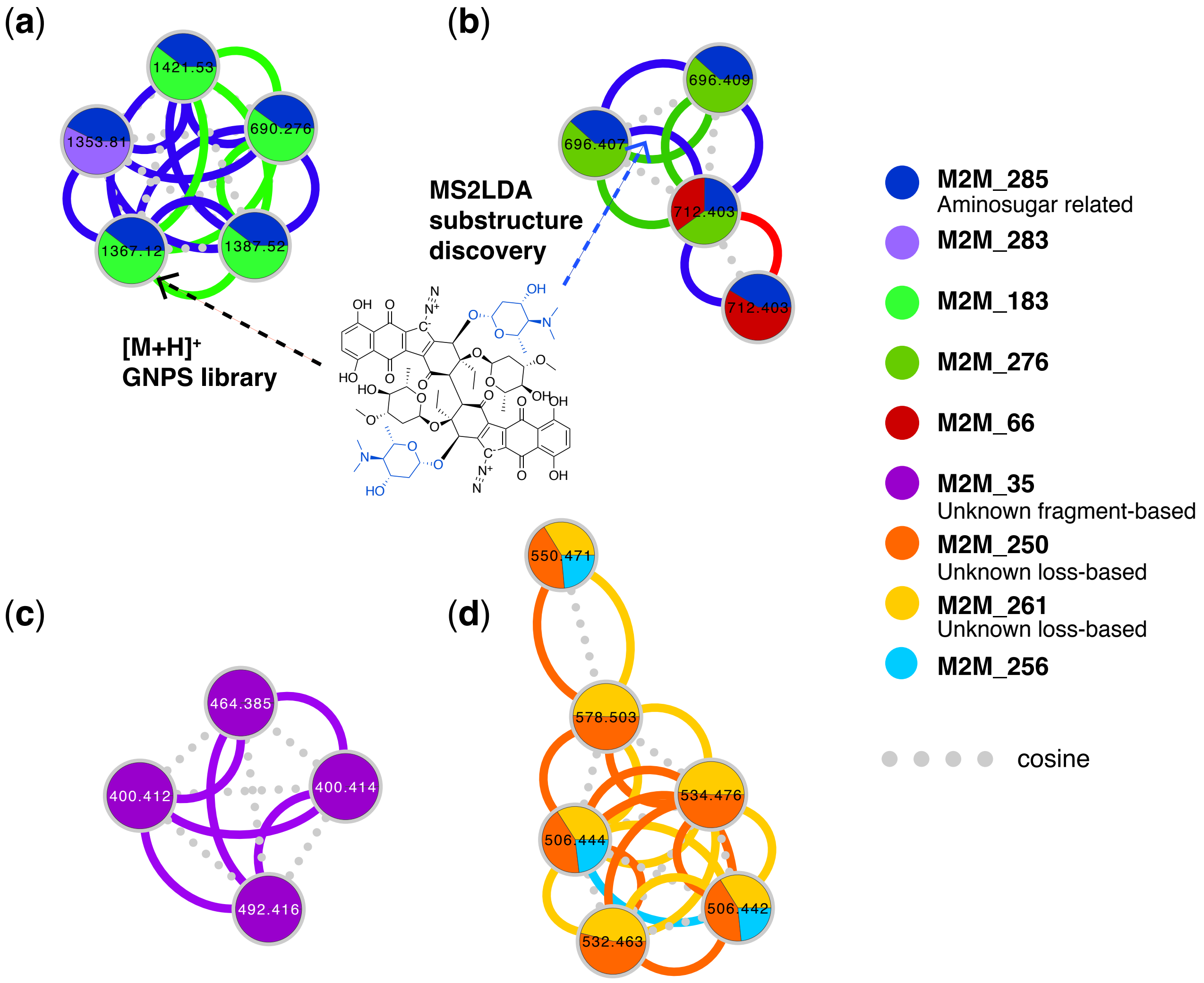
(a)
(b)
(c)
(d)
1421.53
1353.81
690.276
1367.12
1387.52
696.409
696.407
712.403
712.403
MS2LDA
substructure
discovery
[M+H]+
GNPS library
550.471
578.503
534.476
506.444
506.442
532.463
464.385
400.412
400.414
492.416
M2M_285
Aminosugar related
M2M_283
M2M_183
M2M_276
M2M_66
M2M_35
Unknown fragment-based
M2M_250
Unknown loss-based
M2M_261
Unknown loss-based
M2M_256
cosine
