# Supplementary material for: MolNetEnhancer: Enhanced Molecular Networks by Integrating Metabolome Mining and Annotation Tools
Source: Metabolites. 2019 Jul 16;9(7):144. doi: 10.3390/metabo9070144 (PMC6680503; doi:10.3390/metabo9070144)
Supplement: Supplementary file 1 [file metabolites-09-00144-s001.zip › Supplementary Materials/Supplementary Materials/Figure_S5.pdf]

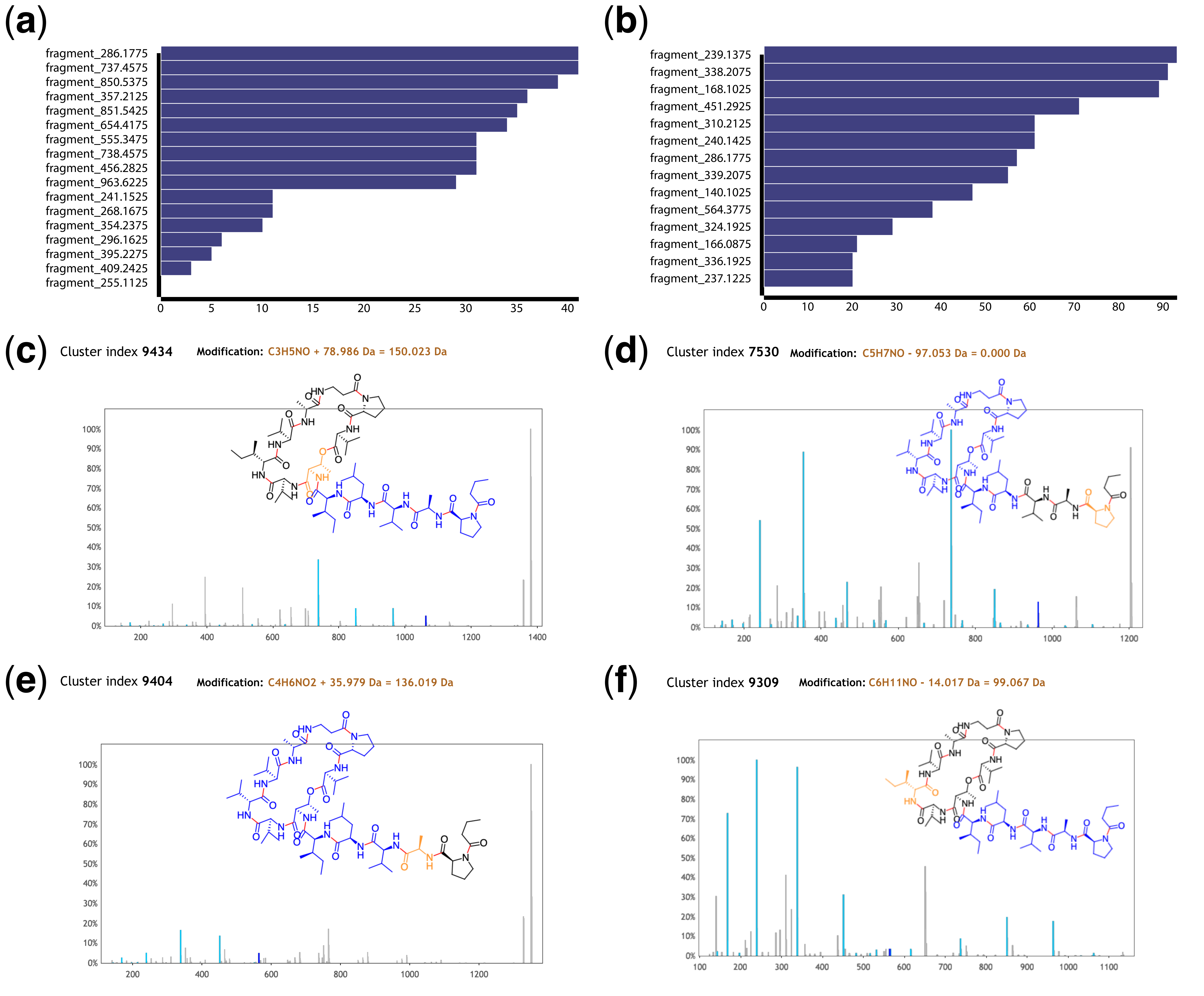
(a)
fragment_286.1775
fragment_737.4575
fragment_850.5375
fragment_357.2125
fragment_851.5425
fragment_654.4175
fragment_555.3475
fragment_738.4575
fragment_456.2825
fragment_963.6225
fragment_241.1525
fragment_268.1675
fragment_354.2375
fragment_296.1625
fragment_395.2275
fragment_409.2425
fragment_255.1125
0 5 10 15 20 25 30 35 40
(b)
fragment_239.1375
fragment_338.2075
fragment_168.1025
fragment_451.2925
fragment_310.2125
fragment_240.1425
fragment_286.1775
fragment_339.2075
fragment_140.1025
fragment_564.3775
fragment_324.1925
fragment_166.0875
fragment_336.1925
fragment_237.1225
0 10 20 30 40 50 60 70 80 90
(c)
Cluster index 9434
Modification: C3H5NO + 78.986 Da = 150.023 Da
(d)
Cluster index 7530
Modification: C5H7NO - 97.053 Da = 0.000 Da
(e)
Cluster index 9404
Modification: C4H6NO2 + 35.979 Da = 136.019 Da
(f)
Cluster index 9309
Modification: C6H11NO - 14.017 Da = 99.067 Da
