# Supplementary material for: MolNetEnhancer: Enhanced Molecular Networks by Integrating Metabolome Mining and Annotation Tools
Source: Metabolites. 2019 Jul 16;9(7):144. doi: 10.3390/metabo9070144 (PMC6680503; doi:10.3390/metabo9070144)
Supplement: Supplementary file 1 [file metabolites-09-00144-s001.zip › Supplementary Materials/Supplementary Materials/Supplementary Figures.pdf]

metabolites

Supplementary Material

MolNetEnhancer: Enhanced Molecular Networks by Integrating Metabolome Mining and Annotation Tools

Madeleine Ernst [1,2*], Kyo Bin Kang [1,3], Andrés Mauricio Caraballo-Rodríguez [1], Louis-Felix Nothias [1], Joe Wandy [4], Christopher Chen [1], Mingxun Wang [1], Simon Rogers [5], Marnix H. Medema [6], Pieter C. Dorrestein [1,7,8] and Justin J.J. van der Hooft [1,6,*]

1 Collaborative Mass Spectrometry Innovation Center, Skaggs School of Pharmacy and Pharmaceutical Sciences, University of California San Diego, La Jolla, CA 92093, USA
2 Department of Congenital Disorders, Center for Newborn Screening, Statens Serum Institut, 2300 Copenhagen, Denmark
3 Research Institute of Pharmaceutical Sciences, College of Pharmacy, Sookmyung Women's University, Seoul 04310, Korea
4 Glasgow Polyomics, University of Glasgow, Glasgow G12 8QQ, UK
5 School of Computing Science, University of Glasgow, Glasgow G12 8QQ, UK
6 Bioinformatics Group, Department of Plant Sciences, Wageningen University, 6708 PB Wageningen, The Netherlands
7 Department of Pediatrics, University of California San Diego, La Jolla, CA 92093, USA
8 Center for Microbiome Innovation, University of California San Diego, La Jolla, CA 92093, USA
* Correspondence: maet@ssi.dk (M.E.); justin.vanderhooft@wur.nl (J.J.J.v.d.H.)

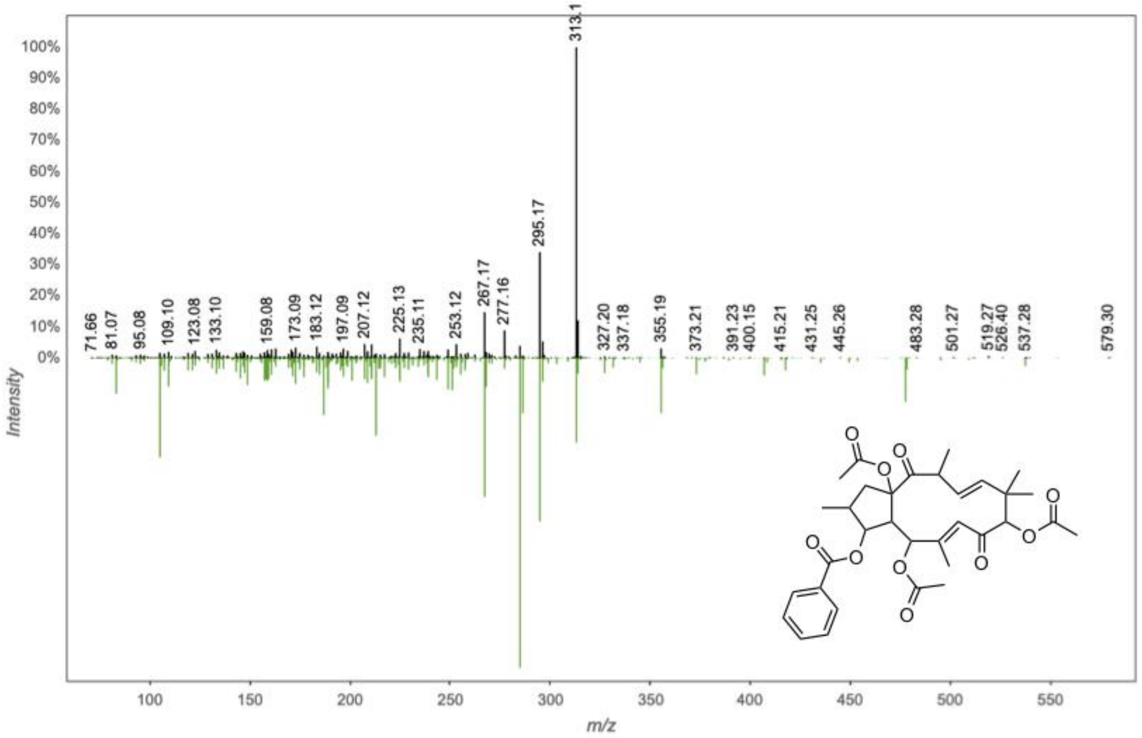

Supporting Figure S1. Mirror plot comparing molecular feature with *m/z* 614.30 and RT 373.17 (black) to GNPS reference spectrum of a jatrophane diterpenoid (green). A total of 289 shared peaks were found. Mass peaks at *m/z* 313, 295, 285 are characteristic for a *Euphorbia* diterpenoid backbone skeleton, however spectral similarity (cosine score) was only found to be 0.71. The unknown molecular feature is thus likely a close structural analogue of the jatrophane diterpenoid. The GNPS reference spectrum as well as the mirror plot is publicly accessible at https://gnps.ucsd.edu/ProteoSAFe/result.jsp?task=26326c233918419f8dc80e8af984cdae&view=view_all_annotations_DB#%7B%22main.%23Scan%23_lowerinput%22%3A%223633%22%2C%22main.%23Scan%23_upperinput%22%3A%223633%22%7D.

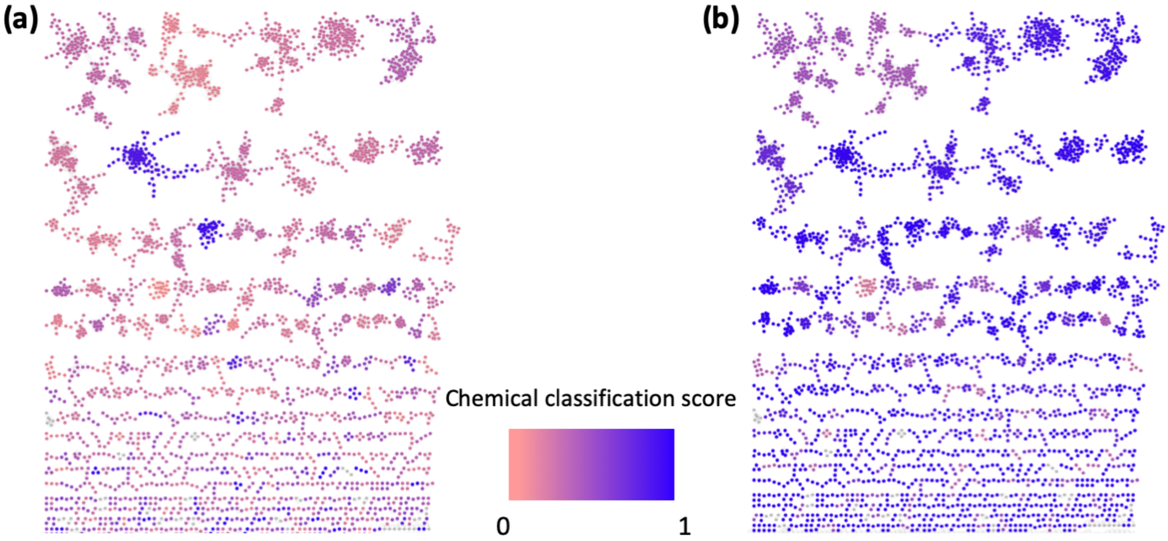

Supporting Figure S2. (**a**) Marine sediment *Salinispora*/*Streptomyces* molecular network colored by chemical classification scores for annotated chemical class terms, and (**b**) same molecular network colored by chemical classification scores for annotated chemical kingdom terms. Light grey means no database matches were found. The higher the class score, the more consistent the chemical annotations are. The kingdom scores represent the database coverage of nodes across a molecular family with scores closer to zero representing families with fewer nodes that have at least one database hit. Whilst most MFs do have database matches for all or most nodes, the consistency in chemical class annotations is - apart from some exceptions - less (indicated by the more orange/pink colors in the left panel). This indicates that for many MF family members the right molecular structures might not yet be present in the structural databases used.

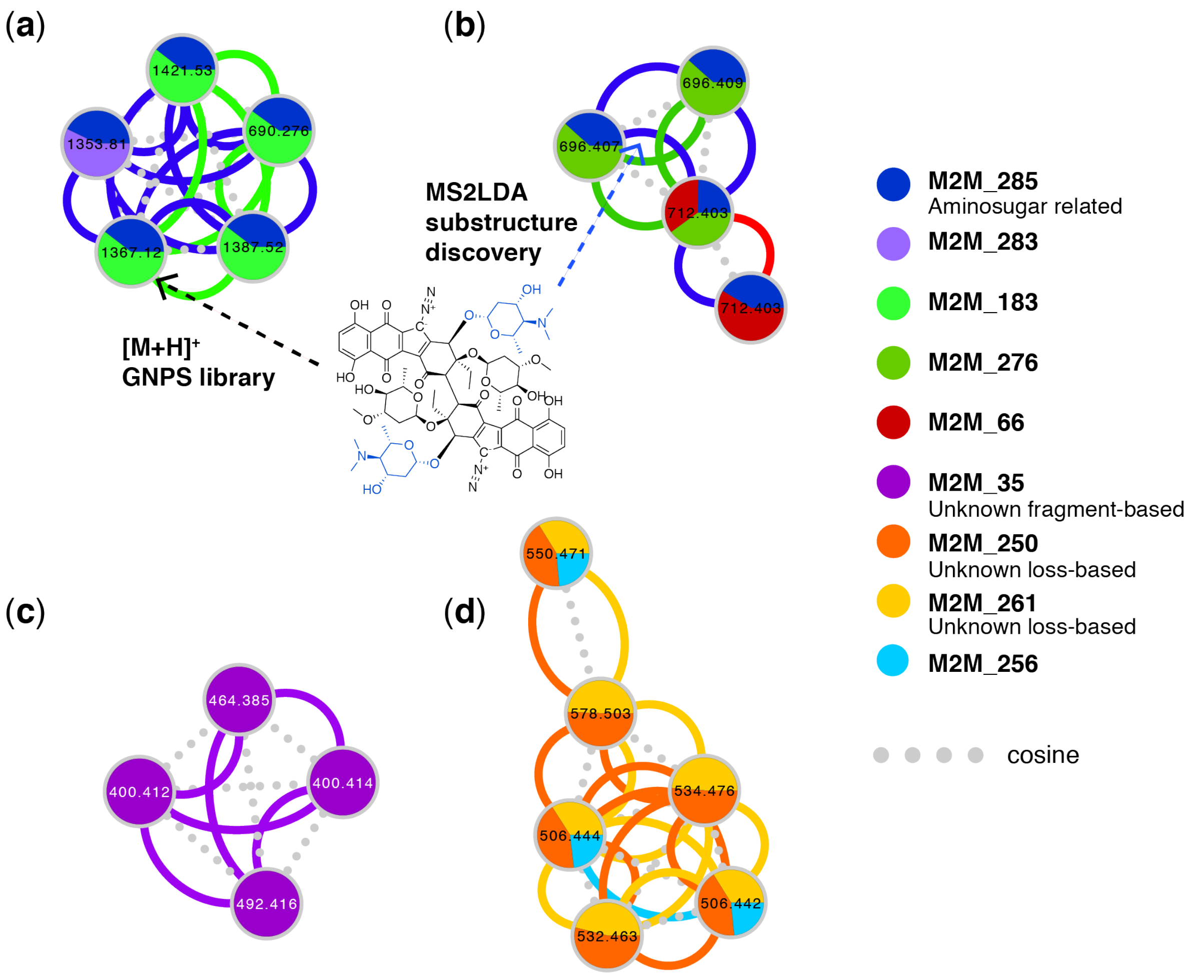

Supporting Figure S3. Molecular families from marine sediment bacteria with color coded Mass2Motif substructure information mapped on them, with (**a**) lomaiviticin-related molecular family where all members contain an amino sugar related motif, (**b**) yet unknown molecular family that shares an amino sugar related motif, (**c**) yet unknown molecular family sharing an unknown fragment-based motif occurring 0.7% in the marine sediment data set, and (**d**) yet unknown molecular family sharing unknown loss-based motifs occurring 0.4% (Mass2Motif 250) and 0.8% (Mass2Motif 261) in the marine sediment data. In all MFs, nodes are colored based on motif overlap scores and the edges present similar colors to show if cosine score-connected nodes share similar Mass2Motifs. It can be seen that in most families multiple motifs are shared across some of its members.

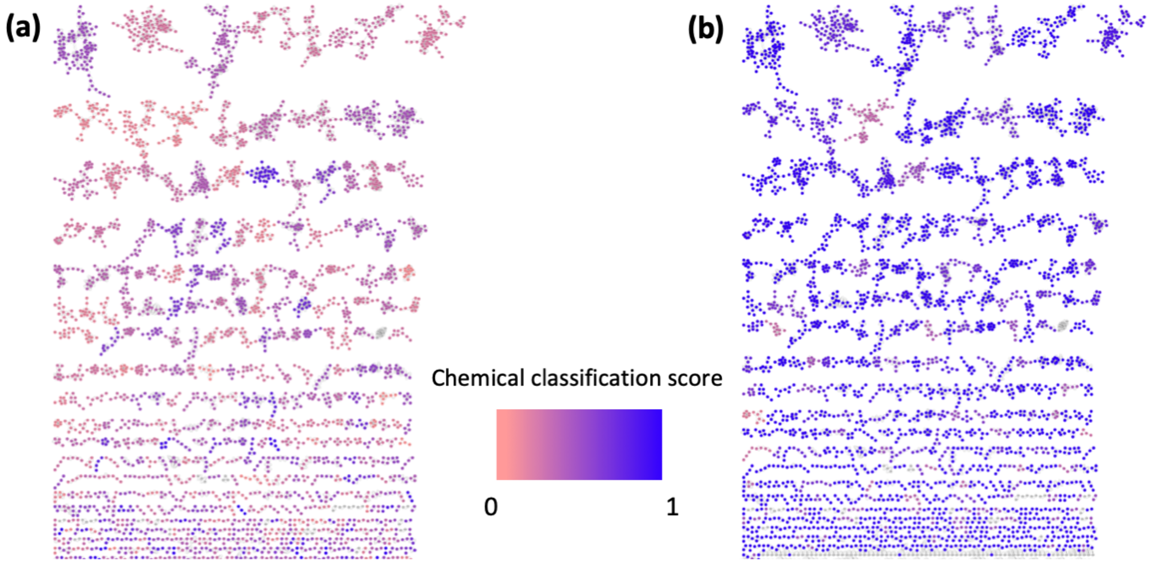

Supporting Figure S4. (**a**) Nematode symbionts *Photorhabdus/Xenorhabdus* network colored by chemical classification scores for annotated chemical class terms, and (**b**) same molecular network colored by chemical classification scores for annotated chemical kingdom terms. Light grey means no database matches were found. The higher the class score, the more consistent the chemical annotations are. The kingdom scores represent the database coverage of nodes across a molecular family with scores closer to zero representing families with fewer nodes that have at least a database hit. We observe database coverages of close to 1 for most molecular families; however, some molecular families have a lower coverage with a few nodes that return candidate structures. Furthermore, we observe that the chemical class annotation is not always consistent indicating that manual inspection and validation of those hits remains essential.

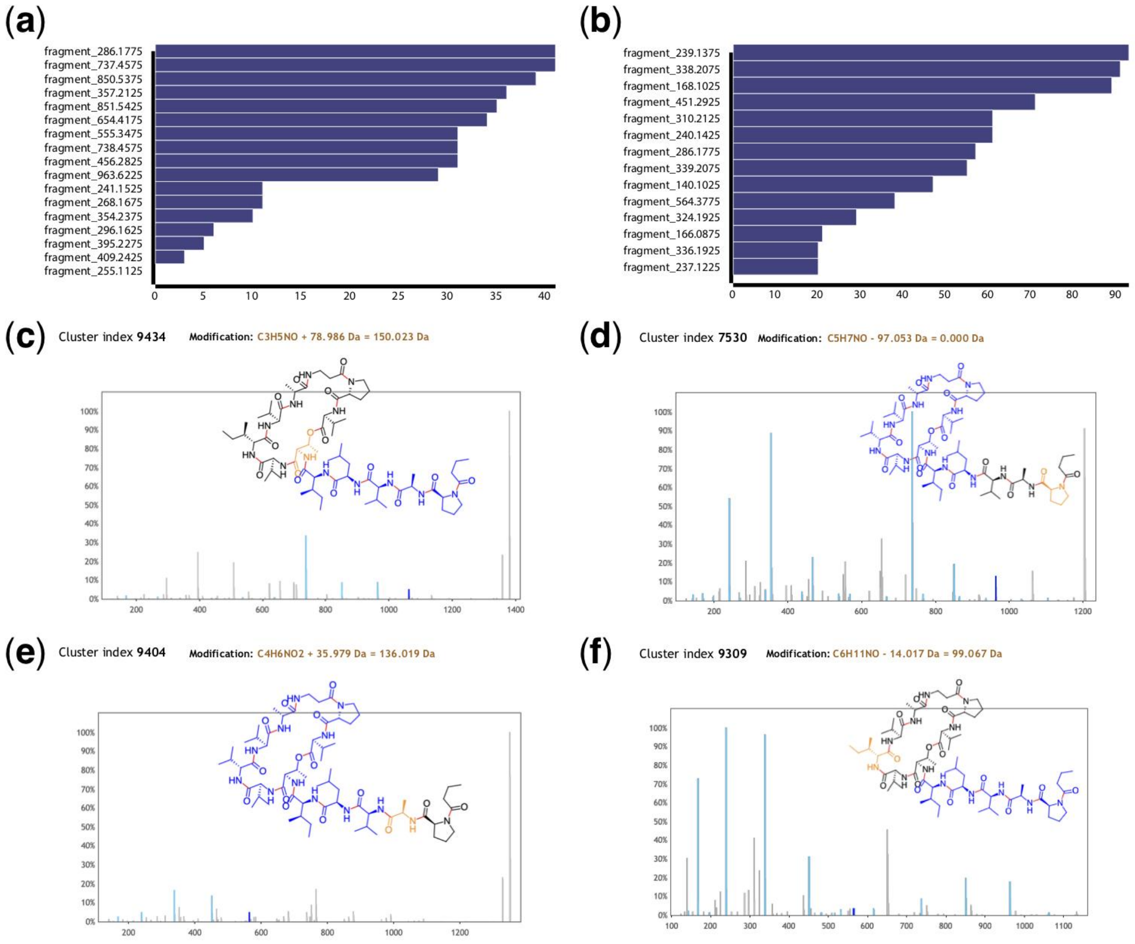

Supporting Figure S5. Xenoamicin Mass2Motif mass feature frequency plots for (**a**) Mass2Motif related to xenoamicin peptidic ring and (**b**) xenoamicin peptidic tail. It can be observed that many mass fragments are present in at least 75% of the associated molecular features (9 and 6 for ring and tail Mass2Motif, respectively) with a few mass fragments present in nearly all associated molecular features. (**c**,**d**) Examples of annotated xenoamicin A modified structures in which only the ring Mass2Motif was found. Indeed, we observe that VarQuest annotates a modified amino acid (addition and loss of) in the tail region of xenoamicin A indicated in orange. (**e**,**f**) Examples of annotated xenoamicin B modified structures in which only the ring Mass2Motif was found. Indeed, we observe that VarQuest annotates a modified amino acid (double water addition, loss of methyl) in the ring region of xenoamicin B indicated in orange. The structures of xenoamicin A and B differ in one methyl group on the amino acid highlighted in orange in (**f**) where B has an isobutyl group and A an isopropyl group. In fact, the structure of xenoamicin A is correctly annotated by VarQuest to this fragmented doubly charged ion.

2019 by the authors. Submitted for possible open access publication under the terms and conditions of the Creative Commons Attribution (CC BY) license (http://creativecommons.org/licenses/by/4.0/).
