# Supplementary figures and images for: MolNetEnhancer: Enhanced Molecular Networks by Integrating Metabolome Mining and Annotation Tools
Source: Metabolites. 2019 Jul 16;9(7):144. doi: 10.3390/metabo9070144 (PMC6680503; doi:10.3390/metabo9070144)

Intensity

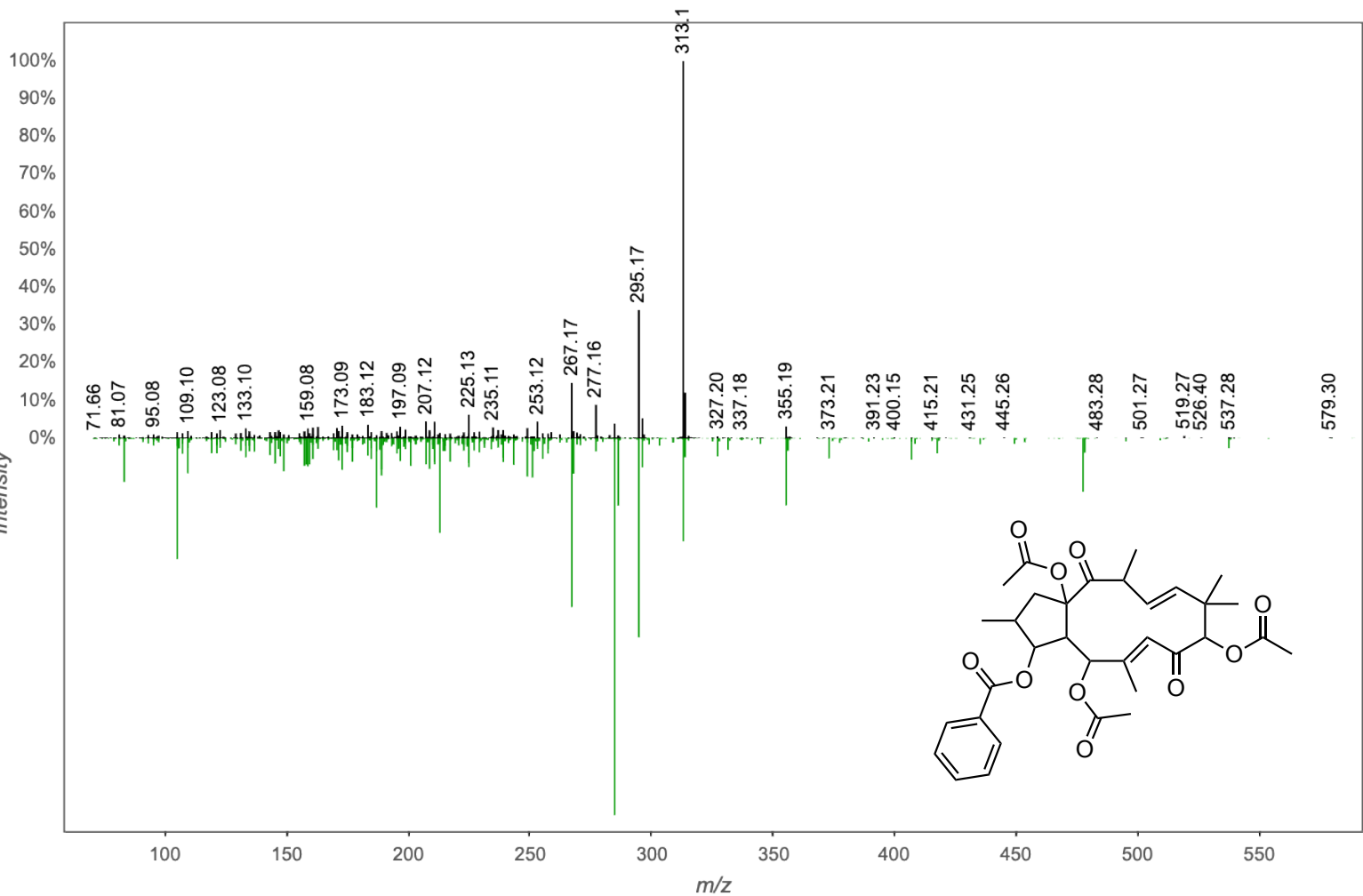

Supplement: Supplementary file 1 [file metabolites-09-00144-s001.zip › Supplementary Materials/Supplementary Materials/Figure_S1.pdf]

**(a)**

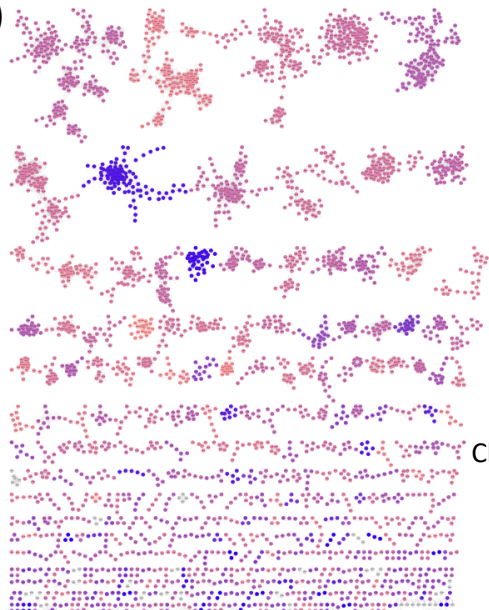

Chemical classification score

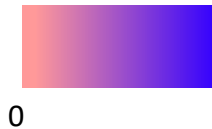

**(b)**

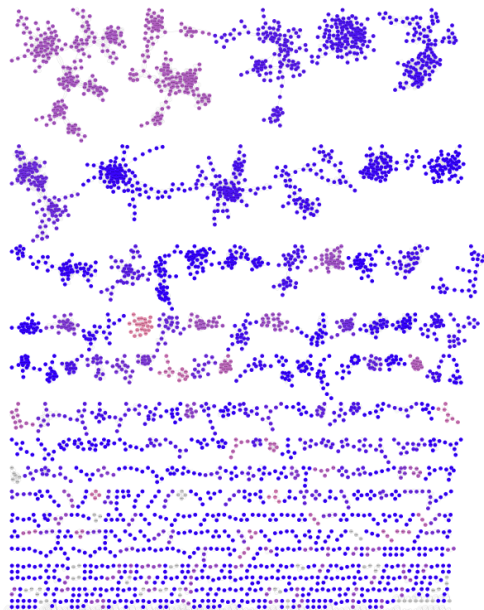

Supplement: Supplementary file 1 [file metabolites-09-00144-s001.zip › Supplementary Materials/Supplementary Materials/Figure_S2.pdf]

**(a)**

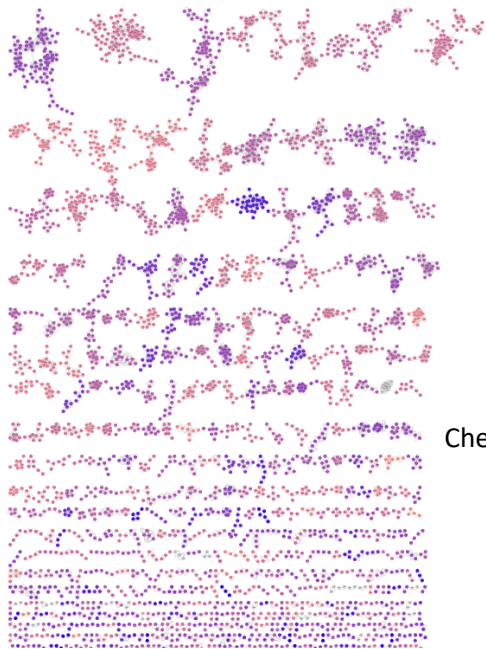

**(b)**

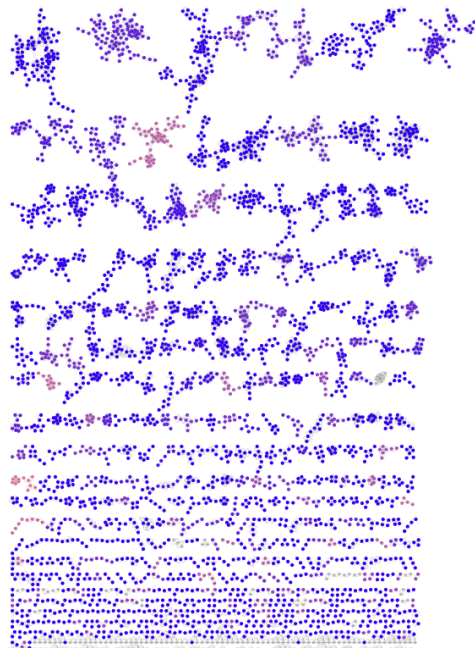

Chemical classification score

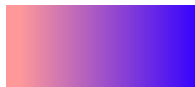

0

1

Supplement: Supplementary file 1 [file metabolites-09-00144-s001.zip › Supplementary Materials/Supplementary Materials/Figure_S4.pdf]
